# Determination of surface proteins profile, capsular genotyping, and antibiotic susceptibility patterns of Group B Streptococcus isolated from urinary tract infection of Iranian patients

**DOI:** 10.1186/s13104-019-4428-4

**Published:** 2019-07-19

**Authors:** Saba Jalalifar, Seyed Asghar Havaei, Tahereh Motallebirad, Sharareh Moghim, Hossein Fazeli, Bahram Nasr Esfahani

**Affiliations:** Department of Microbiology, School of Medicine, Isfahan University of Medical Sciences, Isfahan, Iran

**Keywords:** Antibiotic resistance, Surface protein, Multiplex PCR, Group B *Streptococci*, Urinary tract infection

## Abstract

**Objectives:**

Group B *Streptococcus* (GBS) is an important opportunistic bacteria that causes a wide range of infections including neonatal sepsis, meningitis, pneumonia, soft tissue and urinary tract infections (UTI). The aim of this study was to evaluate the antimicrobial susceptibility patterns, surface proteins and capsular types of GBS isolates.

**Results:**

100 of UTI isolates were confirmed as GBS. Antimicrobial susceptibility pattern showed that 95% of GBS isolates were resistant to tetracycline, followed by erythromycin (52%), clindamycin (47%), levofloxacin (9%) and penicillin, cefepime, cefotaxime, and ceftriaxone each with (8%), and vancomycin 1%. Common capsular types were III, Ib, V, II, Ia and IV respectively and the distribution of surface protein genes was as follows: *rib* (40%), *alpha*-*c* (22%), *alp2/3* (18%) and *epsilon* (15%), and *alp4* gene was not detected in the isolates. Our findings showed the relationship between capsular types with Alpha-like proteins, as well as reduced sensitivity to antibiotics, so the performance of antibiotic surveillance programs is recommended.

**Electronic supplementary material:**

The online version of this article (10.1186/s13104-019-4428-4) contains supplementary material, which is available to authorized users.

## Introduction

*Streptococcus agalactiae*, also known as Group B *streptococcus* (GBS), is a common commensal of the human gastrointestinal and genitourinary tracts [1]. In addition to causing a broad range of infectious diseases in neonates, pregnant women, the elderly and the immunocompromised, this organism is responsible for approximately 2–3% of all urinary tract infections (UTI) [2–4]. Diabetes mellitus, immunocompromised individuals and chronic renal failure may be risk factors for GBS UTI [5, 6]. Therefore the study of the prevalence of GBS infections especially in populations with underlying disease, contributes to epidemiological features of these infections.

With decreased in sensitivity of GBS to Penicillin in recent decade, Macrolides and lincosamides used as alternative therapeutic drugs against GBS [7–9]. Nevertheless, in recent years the resistance of *S. agalactiae* to macrolides, lincosamides and streptogramin B is being reported more often [10]. Hence determining the prevalence of antimicrobial susceptibility pattern of GBS in different regions is important for therapeutic strategy.

The pathogenic mechanisms that underlie acute UTI due to GBS are associated with different virulence factors including Surface-expressed protein adhesion molecules, immune-evasion factors and toxins [11]. The major virulence factors of GBS are the capsular polysaccharide (cps) that is involved in virulence and immune evasion and surface proteins having participated in GBS pathogenesis and immunization.

*cps* type of a GBS strain determines the serotype and these different serotypes have been linked to virulence [12]. This makes it possible to used cps as an epidemiological marker for typing of GBS.

Based on epidemiologic and pathogenic studies, GBS expresses a ten different serotype variants (i.e., Ia, Ib, II, III, IV, V VI, VII, VIII, and IX) capsular polysaccharide that is involved in virulence and immune evasion, which these serotypes differ in their disease-causing abilities [8, 13].

The major surface-localized protein antigens belongs to alpha-like protein (Alp) family and Alpha-C protein, Rib, Alp2, Alp3, Alp4 and the Epsilon protein. They characterized by internal long identical tandem repeats which have an important role in GBS pathogenesis and are considered to be vaccine candidates [8, 14]. Hence, serologic profile of isolates, applied PCR amplification of CPS and surface-localized protein genes of GBS, it is useful to typing the isolates with good discriminatory power and is applicable in diagnosis, treatment and control of this infectious agent.

The aim of this study was to characterize the antibiotic susceptibility patterns, capsular type and surface proteins distribution of GBS isolated from urine samples of patients referring to the educational hospitals of Isfahan, Iran.

## Main text

### Methods

From June 2017 to November 2017 a total of 100 Urine isolates were collected from 2 male and 98 female patients across different age groups who underwent assessment for UTI. The samples that referred in the clinical microbiology laboratory were cultured on 5% sheep-blood agar and incubated overnight at 37 °C (Biolife, Italian). The isolates were characterized phenotypically as *S. agalactiae* by the use of conventional phenotypic and biochemical tests included; Gram staining, colony morphology, catalase, oxidase and CAMP test (Additional file 1) and the confirmed isolates were stored at − 80 °C in brain heart infusion (BHI) broth containing 20% glycerol.

Antimicrobial susceptibility testing was performed based on disk diffusion method on Mueller–Hinton agar with sheep blood (5% v/v) (Himedia, India), in accordance to the Clinical and Laboratory Standards Institute (CLSI) guidelines (2017). Commercially prepared and dehydrated antibiotic discs used in the study were as follows: clindamycin (2 μg), vancomycin (30 μg), erythromycin (15 μg), penicillin (10 units), tetracycline (30 μg), cefepime (30 μg), cefotaxime (30 μg), ceftriaxone (30 μg), levofloxacin (5 μg); disks (MAST, Merseyside, UK). *S. aureus* ATCC 25923 was also used as the quality control. The strain non-susceptible to ≥ 1 antibiotic at least three of antibiotics categories were characterized as MDR.

The MIC for penicillin non-susceptible isolates were determined using the Etest^®^ strip (Liofilchem, Italy 0/002-3 mg/L). The clindamycin-induced resistance was detected in GBS isolates resistant to erythromycin, but susceptible to clindamycin by the double-disc diffusion method (D-zone test) [15].

Chromosomal DNA was extracted using a phenol–chloroform method with some modifications [16]. For the lysis of *S. agalactiae* cells, a pretreatment with higher concentrations of lysozyme (200 mg/ml final concentration) and proteinase K (300 mg/ml final concentration) in the presence of sodium dodecyl sulfate (SDS). The extracted DNA was purified with chloroform-isoamyl alcohol (24:1, vol/vol), phenol–chloroform-isoamyl alcohol (25:24:1, vol/vol/vol) and then precipitated with sodium acetate and ethanol at − 20 °C. Precipitated DNA was washed with 70% ethanol and resuspended in 100 µl of Milli-Q water.

The urine isolates identified phenotypically as *S. agalactiae* were further analyzed to the species confirmation using the PCR amplification of *dlt*S gene.

The identification of capsular genotypes (Ia, Ib and II to IX) of all GBS isolates was performed by multiplex PCR assay as previously described [17]. capsular type of isolates were identified by analyzing the unique banding pattern following agarose gel 1.5% (w/v) electrophoresis and Non-typeable isolates were designated as NT. GBS- specific *dltS* gene was used as internal positive control.

A multiplex PCR assay was applied for direct identification of GBS alpha-protein-like genes, *bca, rib, epsilon, alp 2/3* and *alp4* with five pairs of primers according to the procedure of Creti et al. [14]. In brief, The PCR program was performed by first denaturation at 94 °C for 5 min, followed by 35 cycles of denaturation at 94 °C for 30 s, primer annealing at 56 °C for 30 s and extension at 72 °C for 45 s, and a final extension at 72 °C for 5 min. Positive control was confirmed by direct sequencing of the PCR products of positive GBS isolate.

The SPSS Statistics (IBM SPSS Statistics for Windows, V.20.) was used for statistical analysis. Chi square tests were determined to define possible associations between the variables. *p *< 0.050 were regarded as statistically significant.

### Results

In the present study a total of 100 confirmed *S. agalactiae* isolates were recovered from patients with UTI. A total of 98 GBS isolates (98%) were obtained from female [69 (70.4%) non-pregnant, 9 (9.2%) pre-pregnant and 20 (20.4%) pregnant] and 2% were obtained from male patients.

The results of antimicrobial susceptibility testing based on disk diffusion method indicated that 8% of GBS isolates were resistant to penicillin and MIC of these isolates was confirmed by Etest (non-susceptible, MICs > 0.12). 95% of isolates were resistant to tetracycline, followed by erythromycin (52%) and clindamycin (47%). Also 22% of isolates were recorded as having intermediate resistance to erythromycin and 8% of isolates for clindamycin, while, most of the isolates were sensitive to vancomycin (99%). Simultaneous resistance to both clindamycin and erythromycin was found in 38% of isolates.

Also, the rate of MDR isolates was estimated at 46%. However, the frequency of MDR isolates was higher among penicillin-resistance isolates than penicillin-susceptible but were not significant (100% vs. 41.3%, *p* > 0.05).

The multiplex PCR assay of capsular type GBS revealed that all our isolates were grouped in six serotypes. The GBS capsular serotype isolated from UTI infection in this study were as followed; Serotypes III (n = 41, 41%), serotypes Ib (24%), V (18%), II (7%), Ia (4%) and IV (2%). Only 4% of isolates were initially classified as non-typeable. Serotypes VI, VII, VIII and IX were not detected in this study (Table 1).

**Table 1:** Distribution of the capsular types and *alp* genes among the urinary GBS isolates

Surface alpha-like protein genes No. of isolates (%)	No. of isolates (%) of serotypes						
	Ia n = 4	Ib n = 24	II n = 7	III n = 41	IV n = 2	V n = 18	NT n = 4
*rib* n = 40 (%)	2 (5)	0**	2 (5)	34 (85) **	–	1 (2.5) **	1 (2.5)
*alpha*-*c protein* n = 22 (%)	–	18 (81.8) **	1 (4.5)	0**	–	3 (13.6)	–
*alp 2/3* n = 18 (%)	–	3 (16.7)	1 (5.6)	6 (33.3)	1 (5.6)	7 (38.9) *	–
*epsilon* n = 15 (%)	2 (13.3) *	0*	3 (20) *	0**	1 (6.7)	6 (40) *	3** (20)
*alp4* n = 0 (%)	–	–	–	–	–	–	–
*alpha*-*c protein *+* alp 2/3* n = 3 (%)	–	2 (66.6)	–	–	–	1 (33.3)	–
*alpha*-*c protein *+* rib* n = 1 (%)	–	–	–	1 (100)	–	–	–
NT n = 1 (%)	–	1 (100)	–	–	–	–	–

* p < 0.05

** p < 0.01

Out of 100 isolates, 99% have surface protein antigen genes as follow: *rib* (40%), *alpha*-*c* (22%), *alp2/3* (18%) and *epsilon* (15%), although *alp4* gene was not detected in the isolates. Three isolates were positive for both *alpha*-*c* and *alp2/3* of genes, and one isolate was positive for both *alpha*-*c* and *rib* of genes. Only one GBS isolate did not harbor any of the genes from the alpha family (Table 1). Also a significant relationship was observed between antibiotic resistance, capsular types and surface protein genes (Table 2).

**Table 2:** Distribution of serotype and *alp* genes among urinary GBS isolates across resistant isolates

Serotype	No. of resistant isolates (%)								
	Penicillin n = 8 (%)	Erythromycin n = 52 (%)	Clindamycin n = 47 (%)	Tetracycline n = 95 (%)	Vancomycin n = 1 (%)	Levofloxacin n = 9 (%)	Cefotaxime n = 8 (%)	Ceftriaxone n = 8 (%0	Cefepime n = 8 (%)
*Ia*	–	2 (3.8)	–	4 (4.2)	–	1 (11.1)	–	–	–
*Ib*	2 (25)	22 (42.3)**	23 (48.9)**	24 (25.3)	–	1 (11.1)	2 (25)	2 (25)	2 (25)
*II*	–	3 (5.8)	1 (2.1)	7 (7.4)	–	1 (11.1)	–	–	–
*III*	0*	8 (15.4)**	8 (17)**	40 (42.1)	–	0**	0*	0*	0*
*IV*	–	2 (3.8)	2 (4.3)	1 (1.1)**	–	–	–	–	–
*V*	2 (25)	12 (23.1)	9 (19.1)	16 (16.8)	–	5 (55.6)**	2 (25)	2 (25)	2 (25)
NT	4 (50)**	3 (5.8)	4 (8.5)	3 (3.2)	1 (100)**	1 (11.1)	4 (50)**	4 (50)**	4 (50)**
alp gene									
*rib*	1 (12.5)	8 (15.4)**	8 (17)**	40 (42.1)	1 (100)	1 (11.1)	1 (12.5)	1 (12.5)	1 (12.5)
*alpha*-*c protein*	2 (25)	20 (38.5)**	21 (44.7)**	22 (23.1)	–	1 (11.1)	2 (25)	2 (25)	2 (25)
*alp 2/3*	–	9 (17.3)	9 (19.1)	16 (16.8)	–	–	–	–	–
*epsilon*	3 (37.5)	12 (23.1)*	5 (10.6)	13 (13.7)	–	6 (66.7)**	3 (37.5)	3 (37.5)	3 (37.5)
*alp4*	–	–	–	–	–	–	–	–	–
*alpha*-*c protein *+* alp 2/3*	2 (25)**	2 (3.8)	3 (6.4)	3 (3.2)	–	1 (11.1)	2 (25)	2 (25)	2 (25)
*alpha*-*c protein *+* rib*	–	–	–	0**	–	–	–	–	–
NT	–	1 (1.9)	1 (2.1)	1 (1.1)	–	–	–	–	–

* p < 0.05

** p < 0.01

### Discussion

*Streptococcus agalactiae* has been known, since the 1970s, and it is frequently found in the genitourinary and the gastrointestinal tracts, but it is also the predominant cause of neonatal infection and recently was accounted as an important etiological agent in immunocompromised and elderly patients [2, 18]. The most important factors involved in virulence of GBS are the capsular polysaccharide (cps) and surface protein antigen [15, 19]. Due to the geographical distribution and high diversity of type of the CPS and surface protein antigens, they could be used as an appropriate marker for epidemiology studies [20]. Epidemiological studies have demonstrated the predominance of particular serotypes in the invasive and non-invasive infections, furthermore geographical variations in the distribution of serotypes as well as the emergence of particular clones have been noted [21, 22]. In this field, the knowing the data of circulating serotypes and their characteristics in relation to the virulence properties are accounted an effective instrument in control of infections caused by GBS [23]. According to previous study, the correct identification of the cps types is crucial in the design of a broad coverage glycoconjugate vaccine which would support the most disseminated cps types with GBS surface exposed-antigens, as the Alpha-like proteins that have been demonstrate as promising vaccine candidates [24]. Penicillin is the first-line agents for both prophylaxis and treatment of *S. agalactiae* infections, although several studies have demonstrated the reduced susceptibility to penicillin against GBS infections [25, 26]. These findings demonstrated that frequencies of PRGBS in *S. agalactiae* from Isfahan have some differences from those that have been reported in other geographic regions. According to our observations, 8% of GBS isolates were PRGBS. While, it was higher than reports from Northwest Iran (1.7%) [27], and lower than reports from Seki et al. Japan (14.7%) [28].

In the current study, vancomycin was the most effective antibiotic agent with the susceptibility rate of 99%. Simoes et al. from Brazil and Shabayekt et al. from Egypt, reported susceptibility rate 100% [29, 30]. In our findings, the rate of resistance to tetracycline was 95% which was slightly lower than reports from Brazil (97%) [22] and Tunisia (97.3%) [31]. Our data demonstrated that the resistance rate for both erythromycin and clindamycin were 52% and 47%, respectively. The rates of resistance to erythromycin and clindamycin were higher than Studies conducted in Malaysia [15, 32]. While, in another study carried out by Guoet al. from China, higher resistance was described for erythromycin (63%) and lower resistance was described for clindamycin (44%) [33]. According to the previous findings from Brazil and Malaysia, all of the isolates were susceptible to cephalosporins [1, 15], whereas, our results showed a higher resistance to cephems groups as reports Jannati et al. from Northwest, Iran [27]. This organism expresses capsular polysaccharides that are important virulence factors, epidemiological markers and are the main components of conjugate vaccines. The capsular types of 96% isolates were identified, in this study. Six serotypes (Ia, Ib, II, III, IV, V) were detected and the serotype III was the most common type (41%), which was different to that previously described in Tabriz, Northwest Iran by Nahaei et al. [34] but was similar to those reported in Kashan [35], China [21], Portugal [9] and America [36]. *alp* Genes have an important role in GBS pathogenesis and are also conjugate vaccine candidates with CPS. Approximately most of GBS strains possess one of *alp* genes. Our multiplex PCR analysis results showed that the frequency of *rib* in GBS isolates was 40%, as predominant type that was similar to other geographical regions of Iran, Chinaand Italy [3, 37, 38]. Only 1% of the tested isolates were negative for any existing *alp* genes and 4% were positive for two genes. In our finding, a significant relationship was observed between serotypes and surface protein genes. However, it should be reiterated that the isolates studied in this study, contrary to other studies conducted in Iran, have been related to urinary tract infection.

In summary, our results showed reduced sensitivity to antibiotics, especially against penicillin in *S. agalactiae* isolates which may be related to inappropriate and excessive administration of these antibiotics in patients, therefore, monitoring and control in the administration of antimicrobial agents and better awareness of the source of antibiotic resistance is recommended. In addition, most of our isolates were distributed to capsular serotype III, Ib and V and *rib* and *alpha*-*c* as surface protein antigen genes are more prevalent than other surface protein.

## Limitation

Although the distribution of surface proteins of GBS strains will be useful in epidemiological studies and design of vaccines; but further genetic research on a larger number of GBS isolates is necessary for epidemiological investigation and vaccine development.

## Additional file

**Additional file 1.** CAMP test.

## Data Availability

The datasets used and/or analyzed during the current study are available from the corresponding author on reasonable request

## Abbreviations

SDS: sodium dodecyl sulfate
D-zone test: double-disc diffusion method
CLSI: Clinical and Laboratory Standards Institute
TSB: Tryptic Soy Broth
Alp: alpha-like protein
cps: capsular polysaccharide
ABU: asymptomatic bacteriuria
UTI: urinary tract infections
GBS: Group B *Streptococcus*
