# Supplementary material for: Determination of surface proteins profile, capsular genotyping, and antibiotic susceptibility patterns of Group B Streptococcus isolated from urinary tract infection of Iranian patients
Source: BMC Res Notes. 2019 Jul 19;12:437. doi: 10.1186/s13104-019-4428-4 (PMC6642507; doi:10.1186/s13104-019-4428-4)
Supplement: Supplementary file 1 — Additional file 1. CAMP test. [file 13104_2019_4428_MOESM1_ESM.docx]

CAMP Test

Purpose

The Christie, Atkins, and Munch-Peterson (CAMP) test is used to differentiate group B streptococci (*Streptococcus agalactiae*– positive) from other streptococcal species. *Listeria* *monocytogenes* also produces a positive CAMP reaction.

Principle

Certain organisms (including group B streptococci) produce a diffusible extracellular hemolytic

protein (CAMP factor) that acts synergistically with the beta-lysin of *Staphylococcus* *aureus* to cause enhanced lysis of red blood cells. The group B streptococci are streaked perpendicular to a streak of *S. aureus* on sheep blood agar. A positive reaction appears as an arrowhead zone of hemolysis adjacent to the place where the two streak lines come into proximity.

Method

1.Streak a beta-lysin–producing strain of *S.aureus* down the center of a sheep blood agar plate.

2. Streak test organisms across the plate perpendicular to the *S. aureus* streak within 2 mm. (Multiple organisms can be tested on a single plate).

3. Incubate overnight at 35°-37°C in ambient air.

Expected Results

Positive: Enhanced hemolysis is indicated by an arrowhead-shaped zone of betahemolysis

at the juncture of the two organisms (Figure 1, *A*).

Negative: No enhancement of hemolysis (Figure 1, *B*).

Quality Control

Positive: *Streptococcus agalactiae* (ATCC13813)—enhanced arrowhead hemolysis

Negative: *Streptococcus pyogenes* (ATCC19615)—beta-hemolysis without enhanced arrowhead formation

**Figure 1:** CAMP test. **A,** Positive; arrowhead zone of beta-hemolysis *(at arrow)*, typical

of group B streptococci. **B,** Negative; no enhancement of hemolysis.

1. Reference: Bailey and Scott’s Diagnostic Microbiology.
